# Application of a multiplex immunochromatographic assay for rapid identification of carbapenemases in a clinical microbiology laboratory: performance and turn-around-time evaluation of NG-test Carba 5

**DOI:** 10.1186/s12866-021-02309-9

**Published:** 2021-09-29

**Authors:** Jung Yoon, Chang Hyun Kim, Soo-Young Yoon, Chae Seung Lim, Chang Kyu Lee

**Affiliations:** 1 Department of Laboratory Medicine, Korea University College of Medicine, 126-1, Anam-dong 5-ga, Seongbuk-gu, Seoul, 02841 Republic of Korea; 2 Department of Laboratory Medicine, Korea University Medical Center (KUMC), Guro Hospital, Seoul, South Korea

**Keywords:** Carbapenemase, NG-test Carba 5, Xpert Carba-R, Microbiology laboratory, Turn-around-time

## Abstract

**Background:**

Prompt and accurate identification of carbapenemase production is essential for appropriate treatment and infection control. NG-Test Carba 5 (termed herein “Carba 5”; NG Biotech, Guipry, France) is a multiplex immunochromatographic assay for the rapid phenotypic identification of five major carbapenemases (KPC, NDM, VIM, IMP, and OXA-48-like) from bacterial isolates. This study aimed to evaluate the diagnostic performance of Carba 5 and its impact on the turn-around-time in a clinical microbiology laboratory.

**Results:**

Carba 5 was retrospectively evaluated using 78 carbapenemase producers and 23 non-carbapenemase producers confirmed by PCR and sequencing. The performance and time required for carbapenemase identification were prospectively evaluated using 47 carbapenem resistant Enterobacteriaceae isolates, and the results were compared to those obtained using Xpert Carba-R (Cepheid, Sunnyvale, CA, USA). For the bacterial isolates included in retrospective and prospective evaluation, the Carba 5 assay correctly identified 147 isolates except one isolate with a sensitivity of 99.13% (95% CI 95.25–99.98%) and specificity of 100% (95% CI 89.42–100%). The Carba 5 assay missed one VIM-1 among 13 VIM producers. The assay showed a sensitivity of 92.31% (95% CI 63.97–99.81%) for detecting VIM and 100% for detecting KPC, NDM, OXA-48-like, and IMP. Compared to the Xpert Carba-R assay, Carba 5 exhibited 100% agreement and was more time-efficient (median time 24 min vs. 1 h 11 min).

**Conclusions:**

The Carba 5 assay has potential as an alternative to molecular methods for detecting major carbapenemases from bacterial isolates in a clinical microbiology laboratory. Compared to the Xpert Carba-R, Carba 5 turns out to be more affordable and time-efficient while showing a comparable performance, and may accelerate therapeutic and infection control decisions.

**Supplementary Information:**

The online version contains supplementary material available at 10.1186/s12866-021-02309-9.

## Background

The emergence and spread of carbapenem resistance in gram-negative bacteria, specifically Enterobacteriaceae and *Pseudomonas aeruginosa* species, have become a global health threat [1–3]. Carbapenemase production has the greatest potential for dissemination of carbapenemase-producing isolates by horizontal transfer of plasmids, which frequently possess other resistance determinants. Therefore, prompt and accurate identification of carbapenemase-producing organisms (CPOs) is essential for preventing further spread and infection control [3–5].

Detection and characterization of carbapenemases usually involve phenotypic methods such as the carbapenemase inactivation method (CIM), a modified version of CIM (mCIM), but these methods require overnight culture for carbapenemase detection [6, 7]. For the rapid detection and characterization of carbapenemases in bacterial isolates, several commercialized colorimetric tests have been developed, which provide results in 30 to 120 min. However, they have shown variable performance depending on the carbapenemase activity [8, 9]. Polymerase chain reaction (PCR)-based point-of-care (POC) assays, such as Xpert Carba-R (Cepheid, Inc., Sunnyvale, CA, USA), are available and show excellent performance for the detection of five major carbapenemases [10, 11]. Although PCR-based POC assays take about an hour, it can substantially reduce the turn-around time in carbapenemase detection compared to targeted PCR-based methods.

Recently, a commercial multiplex immunochromatographic assay, NG-Test Carba 5 (termed herein “Carba 5”; NG Biotech, Guipry, France), has been developed. Carba 5 was designed to identify the five major carbapenemases (KPC, NDM, VIM, IMP, and OXA-48-like) within 15 min using bacterial isolates. Several studies have reported good diagnostic performance of Carba 5-based methods [11–14]. However, the time efficiency of Carba 5 and its impact in the clinical setting have not been evaluated. This study aimed to evaluate the diagnostic performance of the Carba 5 assay, including its sensitivity and specificity for the detection of five major carbapenemases, and to evaluate the time efficiency of the Carba 5 assay by analyzing the time required for detecting carbapenemase in a routine microbiology laboratory setting.

To include bacterial isolates with diverse carbapenemases, the diagnostic performance of the Carba 5 assay was retrospectively evaluated using previously characterized isolates. We also performed a prospective evaluation of the Carba-5 assay in comparison to the Xpert Carba-R (version 2.0), a widely used assay for carbapenemase detection, with respect to diagnostic performance and time required for detecting carbapenemases from bacterial colonies in a routine microbiology laboratory setting.

## Results

### Retrospective analysis of the Carba 5 assay

The Carba 5 assay showed an overall sensitivity of 98.72% (95% confidence interval (CI) 93.06–99.97%) and a specificity of 100% (95% CI 85.18–100%) for the identification of five carbapenemases that the assay targeted. The assay showed the excellent performance for the detection of KPC, NDM, IMP, and OXA-48 (100% sensitivity and specificity). It correctly identified all KPC (KPC-2), NDM (NDM-1, − 5, − 7), IMP (IMP-1, − 6), and OXA-48-like (OXA-48, − 181) except VIM. One isolate (VIM-1) among 13 VIM producers was false-negative in the Carba 5 assay, resulting in a sensitivity of 92.31% (95% CI 63.97–99.81%) for VIM detection. Notably, an isolate co-producing NDM and OXA-48-like was identified using Carba 5. The 23 carbapenemase-negative bacterial isolates yielded negative results. An overview of the bacterial isolates collected for the prospective study is shown in **Table S1** and the retrospective evaluation results for Carba 5 are shown in Table 1**.**

**Table 1:** Performance of Carba 5 assay using isolates from the retrospective analysis

Target carbapenemase	Organism group		No. of positive tests / total no. of isolates	Sensitivity (95% CI)	Specificity (95% CI)
	Enterobacteriaceae	*Pseudomonas* species			
KPC			30/30	100 (88.43–100)	100 (94.94–100)
KPC-2	30				
NDM			26/26	100 (86.77–100)	100 (95.20–100)
NDM-1	24				
NDM-5	1				
NDM-7	1				
VIM			12/13	92.31 (63.97–99.81)	100 (95.89–100)
VIM-1	11				
VIM-2	1	1			
OXA-48-like			5/5	100 (47.82–100)	100 (96.23–100)
OXA-48	4				
OXA-181	1				
IMP			3/3	100 (29.24–100)	100 (96.31–100)
IMP-1	1				
IMP-6		2			
NDM + OXA-48-like			1/1	100 (2.50–100)	100 (96.38–100)
NDM-5 + OXA-181	1				
Overall	76	2	77/78	98.72 (93.06–99.97)	100 (85.18–100)
Non-carbapenemase producers	21	2	23		

### Prospective analysis of the Carba 5 assay in comparison to Xpert Carba-R

Among 47 isolates with decreased susceptibility to a minimum of one carbapenem, 78.72% (*n* = 37/47) of isolates were detected with one or more carbapenemases. An overview of the bacterial isolates collected for the prospective study is shown in **Table S2**. The most commonly detected carbapenemase was KPC followed by NDM, which was detected in 86.49% (*n* = 32/37) and 10.81% (*n* = 4/37) of carbapenemase-producing Enterobacteriaceaes (CPEs), respectively. One isolate coproduced KPC and NDM.

Carba 5 detected all KPC- and NDM-producing Enterobacteriaceae without any false positive results, showing 100% sensitivity (95% CI 90.51–100%) and specificity (95% CI 69.15–100%). The Xpert Carba-R assay also showed 100% sensitivity and specificity. The agreement between the Carba 5 and Xpert Carba-R assays was 100% (Table 2).

**Table 2:** Performance of Carba 5 assay and Xpert Carba-R assay using isolates from the prospective analysis

Target carbapenemase	Number of isolates	Carba 5		Xpert Carba-R	
		% sensitivity (95% CI)	% specificity (95% CI)	% sensitivity(95% CI)	% specificity (95% CI)
Class A					
KPC	32	100 (89.11–100)	100 (78.20–100)	100 (89.11–100)	100 (78.20–100)
Class B					
NDM	4	100 (39.76–100)	100 (91.78–100)	100 (39.76–100)	100 (91.78–100)
Class A + B					
KPC + NDM	1	100 (2.50–100)	100 (92.29–100)	100 (2.50–100)	100 (92.29–100)
Overall	37	100 (90.51–100)	100 (69.15–100)	100 (90.51–100)	100 (69.15–100)
Non carbapenemase producers	10				

### Turn-around time evaluation

**Fig. 1:**
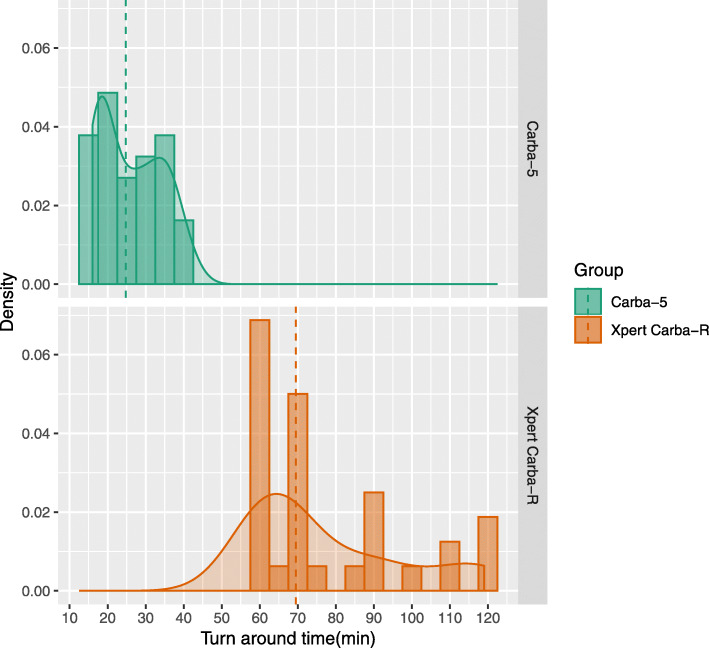
Comparison of turn-around-time between Carba 5 and Xpert Carba-R assays. Density refers to probability density estimated using Kernel density estimation

The median turn-around time evaluation (TAT) of Carba 5 and Xpert Carba-R was 24 min (range 16 min to 42 min) and 1 h 9 min (range 58 min to 1 h 59 min), respectively. The time to the 90th percentile of the TAT was 36 min and 1 h 59 min for Carba 5 and Xpert Carba-R, respectively. Evaluation of the TAT for carbapenemase detection in bacterial isolates showed that the Carba 5 is a significantly more time-efficient method than the Xpert Carba-R (*P* < 0.001). The TATs of Carba 5 and Xpert Carba-R are shown in Fig. 1**.**

### Overall performance of the Carba 5 assay

Based on the overall results including all bacterial isolates from both retrospective and prospective evaluation, the Carba 5 assay yielded a sensitivity of 99.13% (95% CI 95.25–99.98%) and specificity of 100% (95% CI 89.42–100%). The assay showed a sensitivity of 92.31% (95% CI 63.97–99.81%) for detecting VIM and 100% for detecting KPC, NDM, OXA-48-like, and IMP.

## Discussion

The Carba 5 assay is a multiplex immunochromatographic assay designed to identify the five major carbapenemases in bacterial isolates of Enterobacteriaceae and *P. aeruginosa.* In this study, we evaluated the diagnostic performance of the Carba 5 assay using previously collected clinical isolates of Enterobacteriaceae and *P. aeruginosa*. We also prospectively compared the performance and time required for carbapenemase detection with Carba 5 compared to that with Xpert Carba-R. To our knowledge, this is the first study to prospectively evaluate the impact of Carba 5 in a routine microbiology laboratory setting, with respect to the TATs.

In the present study, the Carba 5 assay could identify all isolates producing KPC, NDM IMP, and OXA-48-like but missed one VIM-producing *K. pneumoniae* isolate. We repeated both the Carba 5 assay and culture-based conventional PCR and confirmed this result as a true false negative. Several studies have assessed the performance of Carba 5 assay and revealed high overall sensitivity and specificity [11–14]. Among the five major carbapenemases, the assay showed 100% sensitivity for KPC, VIM, and OXA-48-like in all studies, whereas less to high sensitivities, ranging from 92 to 100%, were obtained for NDM. Detection of IMP using the Carba 5 assay was found to be challenging in several studies [11, 13, 14]. The higher sensitivity obtained in the current study could be due to the small number of isolates included.

In a prospective study, the Carba 5 assay showed comparable performance compared with Xpert Carba-R. Both assays showed 100% agreement for detection of the five major CPEs. Notably, only KPC - and NDM-producing Enterobacteriaceae were included in our prospective study. According to the Korean national surveillance report, among the detected CPEs, the percentages of KPC, NDM, and OXA-48-like genotypes were 74, 17, and 4%, respectively, whereas VIM and IMP were very rare (both less than 2%) [15]; this may explain the limited genotypes of carbapenemases included in our prospective study.

Since rapid detection of carbapenemase is essential for effective therapeutic management and infection control, short TAT is an important aspect of carbapenemase detection methods. In our experience, compared to the carbapenemase inhibition test, both the Carba 5 assay and Xpert Carba-R assay reduced the time required for carbapenemase detection (the time from sample collection to the results reported) by about 29 h (data not shown). The Carba 5 assay was more time efficient compared to Xpert Carba R assay, considering that the median time required for carbapenemase detection was approximately 25% of that required using Xpert Carba-R. In addition, although the price per test varies by country, the Carba 5 assay is more cost-efficient in Korea, as the price per test for the Carba 5 assay is approximately half that of the Xpert Carba-R assay.

The TATs for carbapenemase detection using the Carba 5 and Xpert Carba-R assays could vary depending on the quality control practices of clinical laboratories. In the Xpert Carba-R assay, the results were automatically interpreted by the GeneXpert System. However, taking the importance of carbapenemase detection into account, we manually recheck them through PCR amplification curves of the View Result window before the final report is made. In clinical laboratories that report the automatically interpreted results, the estimated TATs for Xpert Carba-R could be shorter than ours. For the Carba-5 assay, our laboratory retests when a faint band is observed in the control line before reporting negative results. In our experiment, one sample for the Carba 5 assay was retested because a faint band was observed in the control line, which was later confirmed as negative when retested. In addition, pre- or post-analytical steps, such as sample preparation, sample loading, data validation processes may result in wide range of TATs in a clinical laboratory dealing with daily about 450 culture specimens.

This study has limitations in that a relatively small number of CPO isolates, especially isolates producing OXA-48 or IMP, were included. Our data could reflect the performance of Carba 5 in routine microbiology laboratory, particularly in area where KPC and NDM are the major carbapenemases detected. However, further studies with genetically diverse strains are necessary.

## Conclusion

The Carba 5 assay exhibited good performance in detecting five major carbapenemases. Compared to the Xpert Carba-R assay, the Carba 5 assay revealed a comparable performance and was more time-efficient and cost-effective to detect carbapenemase producers in routine microbiology laboratory settings. The Carba 5 assay is thus a powerful method for the rapid detection of CPEs from bacterial isolates and for infection control.

## Methods

### Bacterial isolates

In total, 148 bacterial isolates showing carbapenem resistance were included in the retrospective and prospective analysis, which contained 144 isolates of Enterobacteriaceae and four of *Pseudomonas aeruginosa*. The retrospective isolates were stored at − 80 °C in 20% skim milk. For all isolates, a suspension with 0.5 McFarland turbidity standard from well-isolated colonies was inoculated on MacConkey agar plates and incubated at 37 °C for 16–24 h before testing. This study was approved by the Institutional Review Board of Guro Korea University Hospital (2020GR0390).

The retrospective study was conducted at Korea University Medical Center, Guro Hospital, using a total of 101 clinical gram-negative isolates, including Enterobacteriaceae (*n* = 97) and *P. aeruginosa* (*n* = 4). The collection comprised 23 carbapenemase-negative isolates and 78 CPOs having Ambler classes [16–18] A (*n* = 31), B (*n* = 42), and D (*n* = 5), and one isolate co-producing two carbapenemases (**Table S1**). Each isolate was characterized with targeted PCR and sequencing of carbapenemase, which is considered as reference method for carbapenemase identification in this study.

The prospective study was performed from July to December 2020 at Korea University Medical Center, Guro Hospital. In total, 47 clinical isolates were collected, of which obtained from various types of specimens, including rectal swabs, urine, body fluids, respiratory specimens, and tissues **(Table S2)**. The inclusion criteria included Enterobacteriaceae with decreased susceptibility to imipenem, meropenem, or ertapenem according to the breakpoint cut-off values defined by the CLSI [19]. Carbapenem resistance was initially screened using the Vitek 2 system (bioMeriux, Durham, NC, USA), and disk diffusion testing was performed for confirmation using discs with 10 μg of imipenem, meropenem, and ertapenem (Oxoid, Basingstoke, UK). The diagnostic performance and TAT of the Carba 5 assay and Xpert Carba-R were compared. The presence of carbapenemase was confirmed by targeted PCR.

### Carba 5

**Fig. 2:**
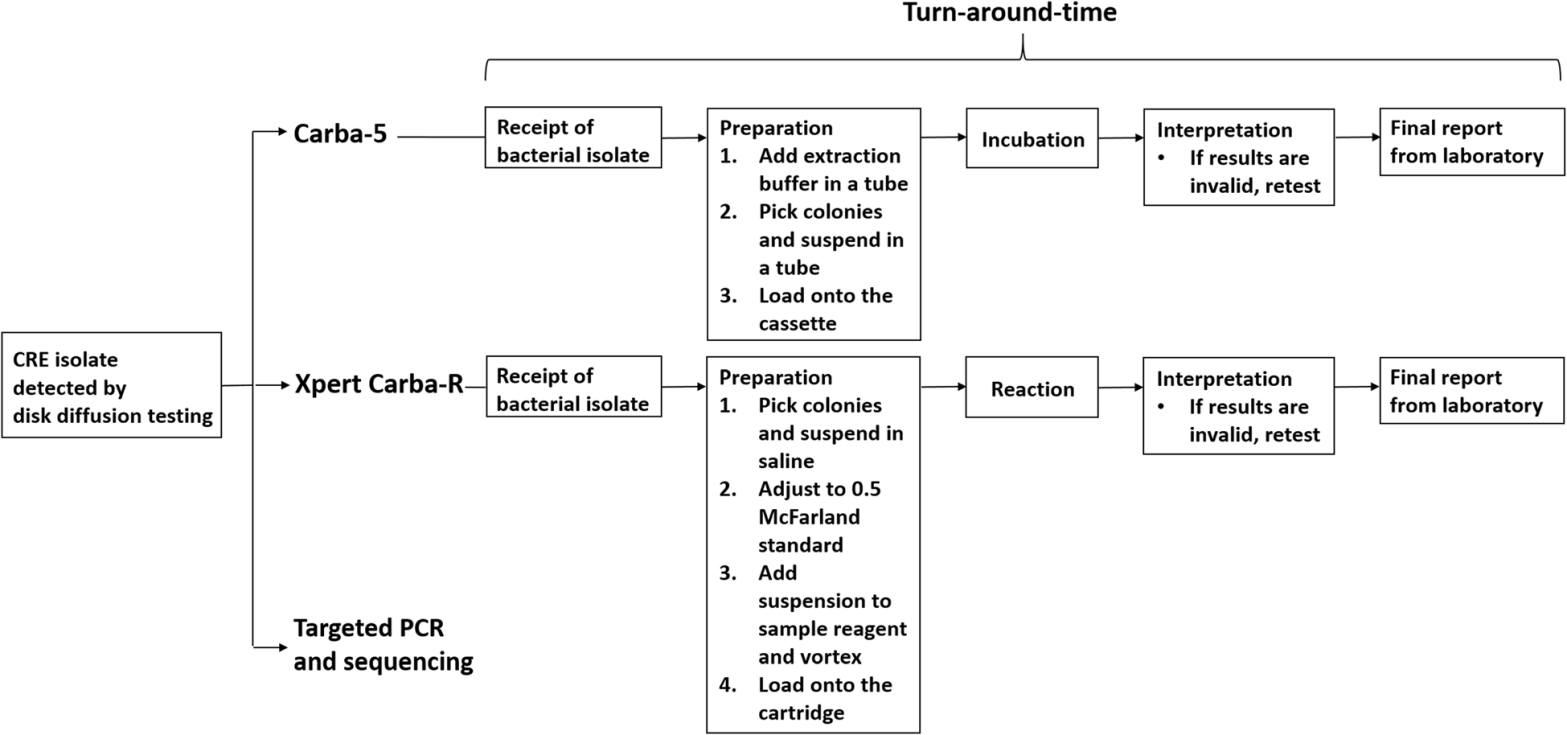
Workflow and turn-around time of Carba-5 and Xpert Carba-R assays

The bacterial isolates grown on MacConkey agar for 16–24 h at 37 °C were tested. Three colonies were picked using a loop and suspended in a microtube provided in the kit, containing 150 μL of extraction buffer. The microtube was vortexed for homogenization. Then, 100 μL of the suspension was loaded onto the Carba 5 device, and the results were visually examined and interpreted after 15 min of incubation (Fig. 2).

### Xpert Carba-R

The Xpert Carba-R assay was performed by 10 μL of bacterial suspension with 0.5 McFarland turbidity standard. Briefly, the bacterial suspension was added to 5 mL of the reagent solution and vortexed. Then, 1.7 mL of the suspension was loaded to the Xpert Carba-R cartridge (Cepheid). The results were automatically obtained and interpreted based on the manufacturer’s recommendation (Fig. 2).

### Targeted PCR and sequencing

All isolates included were screened for the presence of bla_*KPC*_, bla_*NDM*_, bla_*VIM*_, bla_*IMP*_, and bla_*OXA-48*_ by targeted PCR and sequencing using previously reported primers [20, 21]. For confirmation and allele identification of the amplicon, sequencing was performed.

### Turn-around time evaluation

The TAT of Carba 5 assay and Xpert Carba-R were analyzed from September to December 2020. Among the 47 of clinical isolates of the prospective study, 37 isolates were included in TAT analysis. The turn-around time was defined as the time required from the receipt of bacterial isolate until the results were reported in routine clinical practice (Fig. 2).

### Statistics

Data from the retrospective and prospective studies were used to calculate the sensitivity, specificity, and associated 95% CIs, using targeted PCR and sequencing as the reference standard to which the performance of Carba 5 and Xpert Carba-R was compared. For the TAT evaluation, the median value and 90th percentile were calculated. Density distribution of TAT was illustrated using Kernel density estimation. Mann-Whitney U test was performed for continuous variables comparisons, and a *P* value lower than 0.05 was considered significant. All statistical analyses and visualizations were performed using R software (version 3.4.3).

## Supplementary Information

**Additional file 1 Supplementary Table 1.** Details of clinical bacterial isolates included in the retrospective analysis. **Supplementary Table 2.** Details of clinical bacterial isolates included in the prospective analysis.

## Data Availability

The data supporting our findings, such as targeted sequencing data of of bla_*KPC*_, bla_*NDM*_, bla_*VIM*_, bla_*IMP*_, and bla_*OXA-48,*_ are available on the digital repository DataDryad (https://datadryad.org/stash/share/pjFJsOte3KuqekxTbuPMEbY9VZVwlHcYxmn8JZtGzg8).

## Abbreviations

CI: Confidence interval
CIM: Carbapenemase inactivation method
mCIM: A modified version of CIM (mCIM)
CPE: Carbapenemase-producing Enterobacteriaceae
CPO: Carbapenemase-producing organism
ESBL: Extended-spectrum β-lactamase
TAT: Turn-around time
