# Supplementary material for: Application of a multiplex immunochromatographic assay for rapid identification of carbapenemases in a clinical microbiology laboratory: performance and turn-around-time evaluation of NG-test Carba 5
Source: BMC Microbiol. 2021 Sep 29;21:260. doi: 10.1186/s12866-021-02309-9 (PMC8482613; doi:10.1186/s12866-021-02309-9)
Supplement: Supplementary file 1 — Additional file 1 Supplementary Table 1. Details of clinical bacterial isolates included in the retrospective analysis. Supplementary Table 2. Details of clinical bacterial isolates included in the prospective analysis. [file 12866_2021_2309_MOESM1_ESM.docx]

Title page

Application of a multiplex immunochromatographic assay for rapid identification of carbapenemases in a clinical microbiology laboratory: Performance and turn-around-time evaluation of NG-Test Carba 5

Jung Yoon^1^, Chang Hyun Kim^2^, Soo-Young Yoon^1^, Chae Seung Lim^1^, and Chang Kyu Lee^1*^

^1^Department of Laboratory Medicine, Korea University College of Medicine, Seoul, Korea

^2^Department of Laboratory Medicine, Korea University Medical Center (KUMC), Guro Hospital, Seoul, Korea

**Supplementary table 1.** Details of clinical bacterial isolates included in the retrospective analysis

|  | *Klebsiella pneumoniae* | *Escherichia coli* | *Enterobacter cloacae* | *Kluyvera ascorbata* | *Pseudomonas aeruginosa* | *Klebsiella aerogenes* | *Citrobacter freundii* | *Citrobacter koseri* | *Raoultella ornithinolytica* | Others^*^ | Subtotal |
| --- | --- | --- | --- | --- | --- | --- | --- | --- | --- | --- | --- |
| **Class A** |  |  |  |  |  |  |  |  |  |  |  |
| **KPC** |  |  |  |  |  |  |  |  |  |  | **30** |
| KPC-2 | 24 | 3 |  |  |  | 2 |  | 1 |  |  | 30 |
| **Class B** |  |  |  |  |  |  |  |  |  |  |  |
| **NDM** |  |  |  |  |  |  |  |  |  |  | **26** |
| NDM-1 | 8 | 5 | 5 | 4 |  |  | 1 |  |  | 1 | 24 |
| NDM-5 |  | 1 |  |  |  |  |  |  |  |  | 1 |
| NDM-7 |  |  |  |  |  | 1 |  |  |  |  | 1 |
| **VIM** |  |  |  |  |  |  |  |  |  |  | **13** |
| VIM-1 | 11 |  |  |  |  |  |  |  |  |  | 11 |
| VIM-2 |  |  |  |  |  |  |  |  |  | 2 | 2 |
| **IMP** |  |  |  |  |  |  |  |  |  |  | **3** |
| IMP-1 |  |  |  |  |  |  |  |  |  | 1 | 1 |
| IMP-6 |  |  |  |  | 2 |  |  |  |  |  | 2 |
| **Class D** |  |  |  |  |  |  |  |  |  |  |  |
| **OXA-48-like** |  |  |  |  |  |  |  |  |  |  | **5** |
| OXA-48 | 1 | 2 |  |  |  |  | 1 |  |  |  | 4 |
| OXA-181 |  | 1 |  |  |  |  |  |  |  |  | 1 |
| **Class B + D** |  |  |  |  |  |  |  |  |  |  |  |
| NDM-5 + OXA-181 |  | 1 |  |  |  |  |  |  |  |  | 1 |
| **Non carbapenemase  producers** | 13 | 1 | 3 |  | 2 | 1 |  | 1 | 2 |  | **23** |
| **Total** | 57 | 14 | 8 | 4 | 4 | 4 | 2 | 2 | 2 | 4 | 101 |

^*^Others includes one isolate of NDM-1 *Klebsiella oxytoca*, one isolate of VIM-2 *Serratia marcescens,* one isolate of VIM-2 *Pseudomonas putida,* and one isolate of IMP-1 *Enterobacter asburiae.*

**Supplementary table 2.** Details of clinical bacterial isolates included in the prospective analysis

|  | *Klebsiella pneumoniae* | *Escherichia coli* | *Klebsiella aerogenes* | *Citrobacter braakii* | *Enterobacter cloacae* | *Klebsiella oxytoca* | *Citrobacter freundii* | *Serratia marcescens* | Subtotal |
| --- | --- | --- | --- | --- | --- | --- | --- | --- | --- |
| **Class A** |  |  |  |  |  |  |  |  |  |
| KPC | 30 | 1 | 1 |  |  |  |  |  | **32** |
| **Class B** |  |  |  |  |  |  |  |  |  |
| NDM | 1 |  |  | 1 | 1 | 1 |  |  | **4** |
| **Class A + B** |  |  |  |  |  |  |  |  |  |
| KPC + NDM |  |  |  |  |  |  | 1 |  | **1** |
| **Non carbapenemase  producers** | 5 | 3 | 1 |  |  |  |  | 1 | **10** |
| **Total** | 36 | 4 | 3 | 1 | 1 | 1 | 1 | 1 | **47** |
